# DRPLA: understanding the natural history and developing biomarkers to accelerate therapeutic trials in a globally rare repeat expansion disorder

**DOI:** 10.1007/s00415-020-10218-6

**Published:** 2020-10-26

**Authors:** Aiysha Chaudhry, Alkyoni Anthanasiou-Fragkouli, Henry Houlden

**Affiliations:** Department of Neuromuscular Disorders, UCL Institute of Neurology, Queen Square, London, WC1N 3BG UK

## Abstract

Dentatorubral–pallidoluysian atrophy (DRPLA) is a rare neurodegenerative disorder caused by CAG repeat expansions in the atrophin-1 gene and is inherited in an autosomal dominant fashion. There are currently no disease-modifying treatments available. The broad development of therapies for DRPLA, as well as other similar rare diseases, has hit a roadblock due to the rarity of the condition and the wide global distribution of patients and families, consequently inhibiting biomarker development and therapeutic research. Considering the shifting focus towards diverse populations, widespread genetic testing, rapid advancements in the development of clinical and wet biomarkers for Huntington’s disease (HD), and the ongoing clinical trials for antisense oligonucleotide (ASO) therapies, the prospect of developing effective treatments in rare disorders has completely changed. The awareness of the HD ASO program has prompted global collaboration for rare disorders in natural history studies and the development of biomarkers, with the eventual goal of undergoing treatment trials. Here, we discuss DRPLA, which shares similarities with HD, and how in this and other repeat expansion disorders, neurogenetics groups like ours at UCL are gearing up for forthcoming natural history studies to accelerate future ASO treatment trials to hopefully emulate the progress seen in HD.

## Current understanding of DRPLA

Dentatorubral–pallidoluysian atrophy (DRPLA) is a rare autosomal dominant neurodegenerative disorder, characterized by progressive cerebellar ataxia, myoclonus, epilepsy, dementia, choreoathetosis, and psychiatric symptoms [90]. The condition was first described by Titica and van Boegard in 1946, whereby two cases in a family with progressive choreoathetosis, ataxia, and dementia were reported [88]. The term “hereditary DRPLA” was later coined by Naito and Oyanagi in 1982 [61]. DRPLA is classified within the spinocerebellar ataxia (SCA) group, which represents a heterogeneous group of > 40 autosomal dominantly inherited diseases [44]. DRPLA is caused by a CAG-polyglutamine (polyQ) repeat expansion. Nine such polyQ diseases have currently been identified in humans, including Huntington’s disease (HD), spinal and bulbar muscular atrophy (SBMA), SCA 1, 2, 3, 6, 7, and 17 [82].

**Fig. 1:**
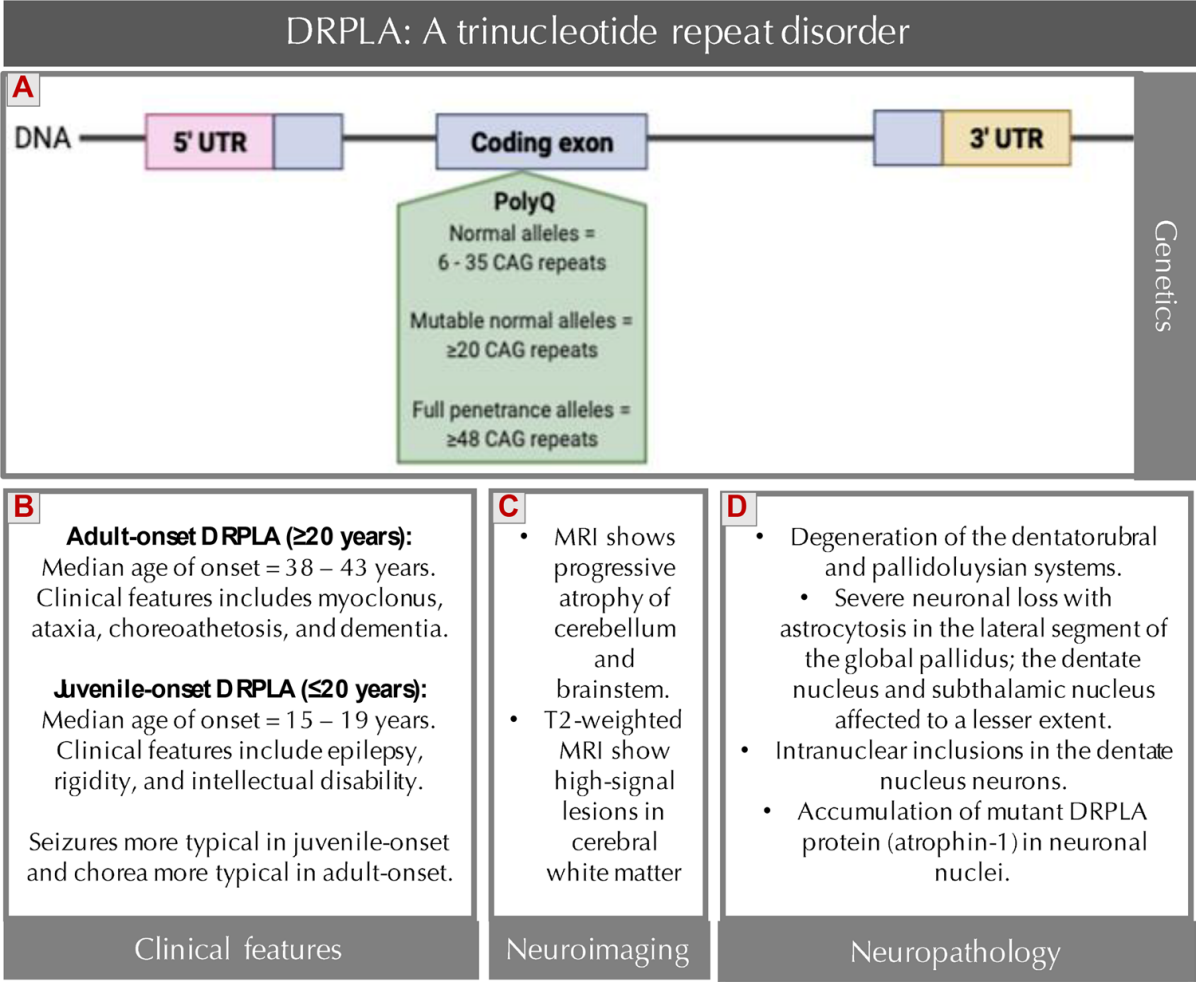
Main genetic, clinical, neuroimaging and neuropathological features of DRPLA. **a** *Illustration of location of the trinucleotide repeat within the gene*—In DRPLA, the CAG trinucleotide repeat expansion occurs in coding exon 5 of the atrophin-1 (*ATN1*) gene, which is located on chromosome 12p13.31. Normal alleles in the *ATN1* gene have 6–35 CAG repeats. ≥ 20 repeats are considered normal mutable alleles that expand on transmission and result in symptoms in the next generation, and ≥ 48 repeats demonstrate fully penetrant clinical phenotype. The unstable CAG repeat sequence causes a polyglutamine (polyQ) expansion in the atrophin-1 protein [13, 25, 44, 55, 60, 68, 94]. **b** *Clinical features*—The primary clinical features of DRPLA are ataxia and cognitive impairment, however, the age of onset affects the clinical presentation, with different symptoms observed between adult-onset and juvenile-onset DRPLA (Wardle et al. [98]; [34, 48, 55]. **c** *Neuroimaging findings*—[47, 106]. **d** *Neuropathological findings—*[36, 84, 106, 107]

DRPLA is the result of an unstable CAG repeat expansion in exon 5 of the atrophin-1 (*ATN1*) gene [46, 60]. The number of repeats in normal individual chromosomes ranges typically between 6 and 35. Full penetrance occurs at ≥ 48 CAG repeats, whilst alleles of 35–47 repeats are incompletely penetrant and are usually associated with a milder clinical phenotype [13, 37, 38, 46, 55, 60]. Characterized by genetic anticipation, with paternal transmission resulting in more prominent anticipation than maternal transmission, DRPLA symptoms present more severely and earlier in each subsequent generation [56, 90]. The CAG repeat load is also associated with the phenotype, whereby the longer the size of expanded CAG repeats, the earlier the age of onset and death, the more severe the symptoms and long-term disability, and the poorer the prognosis [34, 37, 55]. Figure 1 illustrates the currently known features of DRPLA.

Due to the heterogeneity in clinical presentation, based on the prominent genetic anticipation and age of onset, diagnosing DRPLA can often be challenging, with symptoms associated with a broad differential diagnosis. Whilst epileptic seizures are common in juvenile-onset patients (onset prior to the age of 20), the frequency of seizures is reduced after the age of 20, and rare in patients with an onset after the age of 40. Patients with an onset after the age of 20 tend to present with cerebellar ataxia, choreoathetosis and dementia, often making the disease difficult to differentiate from clinical mimics including HD and other hereditary SCAs [13, 61]. Further, brain MRI findings in DRPLA are variable, with case reports of early-stage patients often presenting with only mild changes, whilst late stages of the disease are associated with non-specific changes such as atrophy of the cerebellum and brainstem, complicating the differentiation of the disease from other neurological disorders [35, 45, 77, 83, 92].

## Global burden

**Fig. 2:**
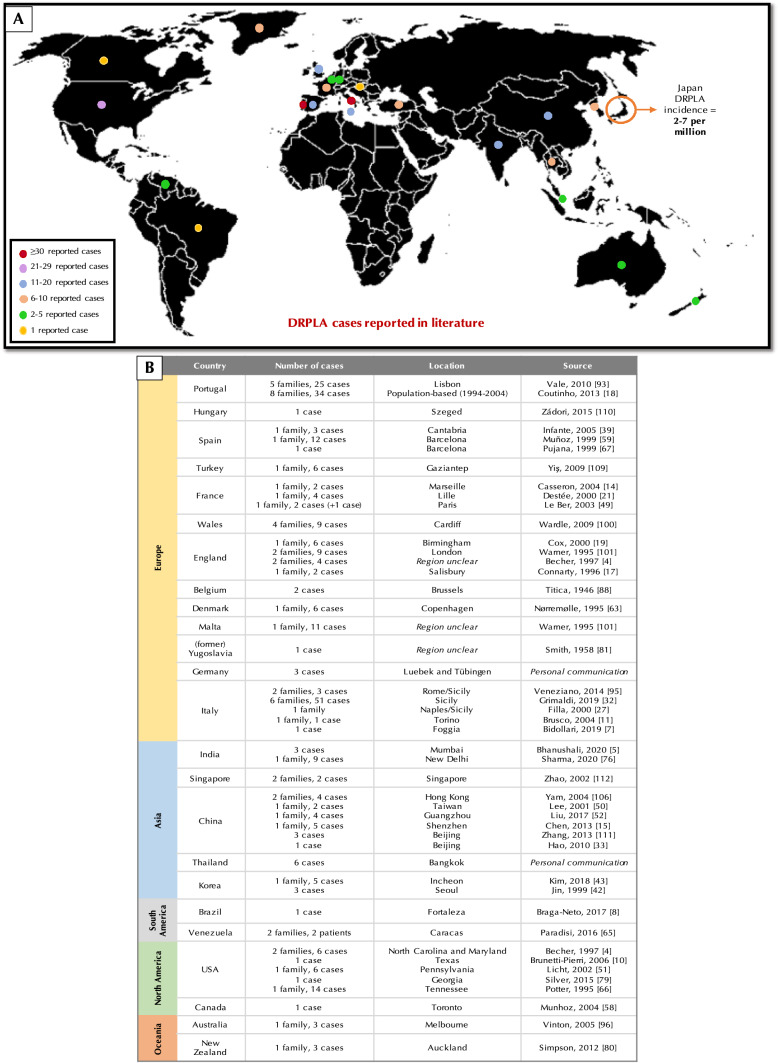
Estimated number of reported cases of DRPLA based on a literature search. **a** World map with dots corresponding to the number of reported cases of DRPLA, as per studies shown in (**b**). **b** Table of DRPLA families/cases found outside of Japan as reported in literature. DRPLA literature search for studies written in English published between January 1945 and July 2020 was performed on PubMed and Scopus databases, using the following key words: DRPLA; dentatorubral–pallidoluysian atrophy; Naito-Oyanagi disease; Haw-River syndrome; or ataxia. Cases for Thailand and Germany based on personal communication

Defining global burden through natural history studies is important to understand the impact of condition and to identify disease biomarkers in the preparation for therapeutic trials. DRPLA is most commonly recognised in populations of Japanese ancestry and has an estimated incidence in Japan of 2–7 per million [26, 71]. DRPLA is considered to be the third most common autosomal dominant ataxia in the Japanese population, accounting for approximately 7.3–20% of autosomal dominant SCA [54, 87, 91]. Whilst it is believed to be rare in non-Asian populations, there are no accurate reports on the worldwide prevalence of DRPLA, with current estimates based on the evaluation of cohorts diagnosed with SCA, suggesting that the prevalence of DRPLA is likely to be underestimated [6, 91, 96]. In Singapore, Korea and China, the frequencies of DRPLA have been found to be 3.4%, 3.4%, and 1%, respectively [42, 50, 111]. In South America, the DRPLA frequency has found to be 0.14% and 3.1% in SCA cohorts from Brazil and Venezuela, respectively [8, 65]. In Europe, findings have been variable, with reports of the frequency in Portuguese cohorts with autosomal dominant ataxias of DRPLA ranging from 4 to 11.2%, whilst in Spain, the frequency was reported as 3.3% [18, 39, 93]. In South Wales, France, and Italy, the frequency amongst cohorts with SCA has been reported as 5%, 0.25%, and 0.45–1%, respectively [11, 27, 49, 97]. Figure 2 illustrates the estimated number of cases around the world.

### Developing a treatment approach for DRPLA and other repeat expansion disorders: drawing upon insights observed for HD

**Fig. 3:**
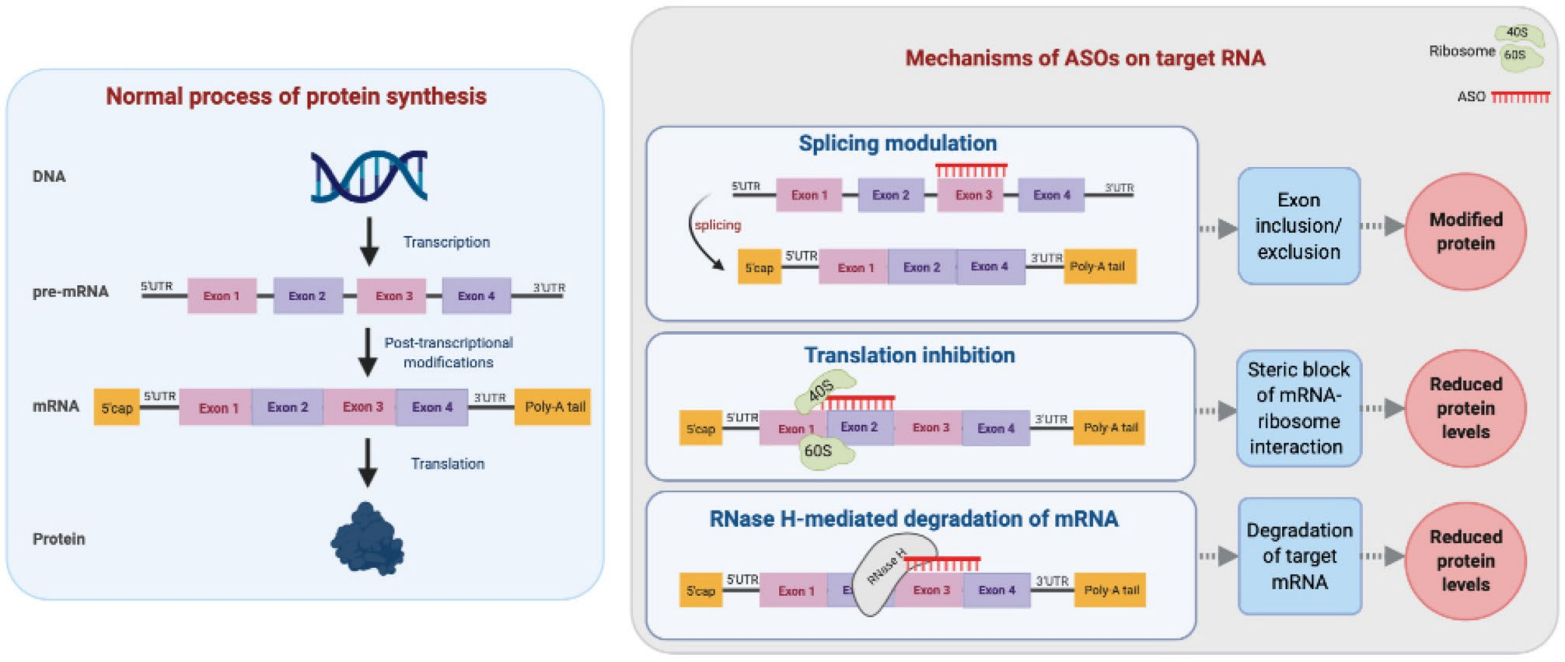
Normal process of protein synthesis and common mechanisms of ASOs on target RNA. *Normal process of protein synthesis* (left): DNA is transcribed to pre-messenger RNA (pre-mRNA), which contains coding (exon) and non-coding (intron) regions between 5′ and 3′ untranslated regions (UTR). Pre-mRNA undergoes post-transcriptional modifications into mature mRNA, including 5′ capping, removal of introns (splicing), and polyadenylation (poly-A tail). The mature mRNA undergoes ribosome-dependent protein synthesis. *Mechanisms of ASOs on target RNA* (right): Splicing modulation—ASO binds to pre-mRNA intron/exon junctions and modulate splicing to include or skip the target exon, resulting in the synthesis of a modified protein. Translation inhibition—ASO binds to the mRNA and sterically blocks and prevents the binding of ribosomes to the mRNA, inhibiting translation and resulting in reduced protein synthesis. RNase H-mediated degradation of mRNA—ASO binds to the mRNA to form an RNA–DNA hybrid, allowing the recruitment of RNase H nuclease, inducing degradation of the target mRNA, resulting in reduced protein synthesis [22, 70, 73, 78, 102]. Diagram created on *biorender.com*

**Fig. 4:**
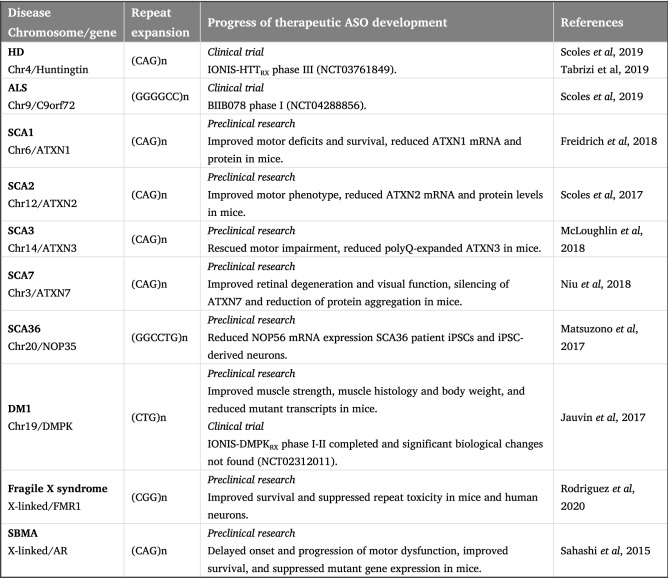
Progress in the development of ASO therapies for repeat expansions associated with neurological disorders. Table shows stages in the research towards developing ASO therapies for neurological repeat expansion disorders. Information. Chr: chromosome; HD; Huntington’s disease; ALS: amyotrophic lateral sclerosis; SCA: spinocerebellar ataxia; DM1: myotonic dystrophy; SBMA: spinobulbar muscular atrophy

There are currently no treatments to prevent or stop the disease progression in DRPLA [90]. Whilst the exact pathophysiology of DRPLA is unclear, the literature overall points towards the idea that the expanded polyQ stretch leads to a “gain-of-toxic” function of the mutant protein on neuronal cells [89]. To downregulate the levels of the pathological polyQ proteins, RNA-targeting therapies may hold promise in the treatment of DRPLA, in particular, antisense oligonucleotides (ASO) therapy [16]. Therapeutic ASOs are single-stranded synthetic DNA molecules that work by binding to complementary target mRNA through Watson and Crick hybridization to interfere with normal gene expression and protein synthesis. ASOs affect gene expression through three mechanisms: RNase H-mediated degradation of mRNA, blocking ribosomes from binding to mRNA and preventing protein translation, or by modulating splicing of pre-mRNA [70, 102]. Figure 3 shows the normal steps of gene expression and the mechanisms by which therapeutic ASOs can influence this process. The scope of ASO therapeutics has expanded considerably in recent years, with an emphasis particularly placed on rare untreatable conditions, which cannot be easily addressed with small molecule drugs. ASO therapeutics have shown promise in several neurological disorders. For example, Nusinersen and Eteplisren are FDA-approved ASO treatment options for spinal muscular atrophy (SMA) and Duchenne muscular dystrophy (DMD), respectively, whilst clinical trials are ongoing for ASO treatments for amyotrophic lateral sclerosis (ALS), Alzheimer’s disease (AD), and HD [75, 104]. Figure 4 highlights the progress of therapeutic ASO development for repeat expansion neurological disorders.

HD is the most widely studied CAG repeat expansion disorder and has gained significant attention for ASO therapeutics. Based on the success seen in preclinical studies of non-human animals, ASO clinical trials are ongoing for HD [78]. HTT_RX_ is an ASO that targets the mutant and wild-type alleles with the purpose of reducing levels of the mutant Huntingtin protein (mHTT). Through phase 1–2a clinical trial in early-stage HD patients, it was found that CSF mHTT levels showed dose-dependent decrease by up to 40%. No significant safety concerns were reported, though levels of CSF neurofilament light chain (NfL), a marker of neuroaxonal damage, were shown to be increased in the final study visit [86]. Preclinical studies have also been conducted for SCA. In early manifest transgenic SCA3 mice, *ATXN3*-targeting ASO resulted in sustained reduction of polyQ-expanded ATXN3, accompanied by rescued motor impairment [57]. Further, in SCA2 mouse models, the delivery of *ATXN2*-targeting ASO led to the downregulation of the *ATXN2* mRNA and protein, delayed onset of the SCA2 phenotype, with improved motor performance [74]. These findings indicate a promising proof-in-concept for ASO therapy as an approach for polyQ conditions.

The advances in HD are suggestive of the possibility of adopting similar methods to define biomarkers and treat DRPLA. Before clinically meaningful interventions can be discovered, a greater understanding of DRPLA disease progression and the identification of wet biomarkers must be pursued. Despite the significant advancements made for other neurodegenerative diseases, such as HD and AD, biomarkers in biological fluid, such as blood and CSF, have not been found for DRPLA [16]. Potential biomarkers including glial fibrillary acidic protein, DJ-1, and tau have been studied in SCA1, SCA2, and SCA6 patients, where only CSF tau was significantly higher in patients than controls, though levels did not correlate with CAG repeat size and disease severity [9]. CSF and plasma/serum NfL have been shown to be a notable biomarker in many neurodegenerative conditions, including HD, AD, ALS, and multiple sclerosis [12, 23, 30, 101]. In a small cohort of repeat-expansion SCA patients, serum NfL was found to be higher in patients than controls; however, the correlation with disease severity was not analysed [103]. Another potentially important biomarker for DRPLA disease progression and severity, which may also act as a potential therapeutic target, is repeat expansion somatic instability. This has been observed in HD mouse models and human brain tissue; it is worth exploring as a prominent biomarker for DRPLA and other repeat expansion disorders [3, 24, 85]. In addition to being clinically beneficial in regard to improving diagnostic accuracy and monitoring disease progression, biological biomarkers for DRPLA would also be influential in research. For example, they would provide greater accuracy in clinical trial recruitment, objective monitoring of disease-related biological changes, tracking adverse effects and response to treatment interventions [1]. Studies in larger cohorts are needed to gather data on the role of biological biomarkers DRPLA.

**Fig. 5:**
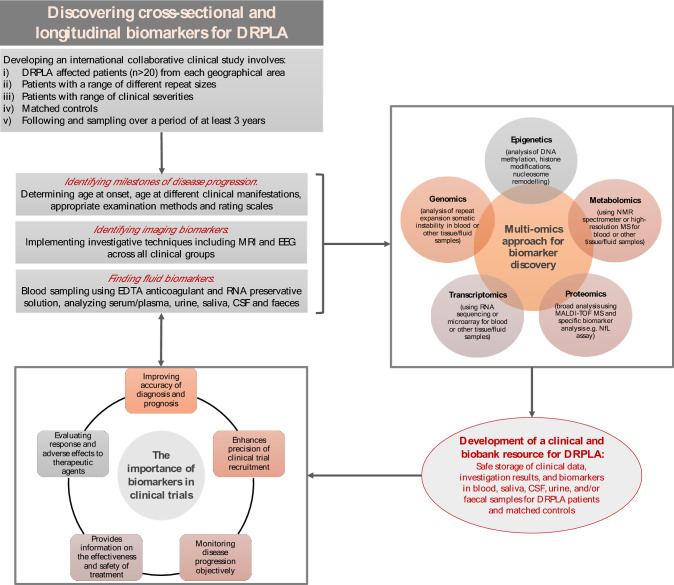
Facilitating DRPLA therapeutic development through understanding of natural history and discovery of biomarkers. Natural history studies follow the course of a disease from prior to inception, through the presymptomatic and clinical stages, to the point it ends (the patient is either cured, chronically disabled or dead, without external intervention) [20]. International, collaborative clinical studies are paramount to the DRPLA drug discovery process by identifying milestones of the disease progression and facilitating the discovery of longitudinal or cross-sectional biomarkers to objectively track disease-related biological changes. The discovery of biomarkers is, in turn, essential for clinical trials. The figure showcases the process by which a clinical and biobank resource for DRPLA can be uncovered, through collaborative efforts. Several methods are used for biomarker discovery; in particular, ‘omics’ technologies contribute towards the rapid discovery and validation of biomarkers. Genomics allows the identification of gene mutations or polymorphisms; transcriptomics can identify changes in RNA; epigenetics can identify modified epigenetic mechanisms; metabolomics and proteomics can identify small molecule metabolites and protein biomarkers in human biological fluid, respectively [1, 2, 28, 31, 41]. MS: multiple spectrometry; NfL: neurofilament light chain; NMR: nuclear magnetic resonance; MALDI-TOF MS: matrix-assisted laser desorption/ionization time of flight mass spectrometry; MRI: magnetic resonance imaging; EEG: electroencephalogram; EDTA: Ethylenediamine tetraacetic acid; CSF: cerebrospinal fluid

Insight into the natural progression of rare diseases is an essential step in facilitating the process of drug development [64]. To garner progress towards the discovery of disease-modifying treatments for DRPLA, emphasis must be placed on natural history studies to enhance our understanding of disease progression and to identify reproducible, validated biomarkers (Fig. 5). For rare diseases, this entails international collaboration to understand the longitudinal clinical progression in statistically large numbers of cases, ideally with *n* > 20 patients from multiple geographical areas, alongside matched controls. Initial investigative markers of disease progression would include clinical rating scales, imaging techniques and EEG, and objective, fluid-based biomarkers. In DRPLA, and other rare repeat expansion disorders, prominent analysis will comprise of DNA extracted from multiple fluids to investigate somatic instability, RNA extracted from blood and fibroblast cell lines, and extraction of serum/plasma and/or CSF for the examination of biomarkers such as NfL. The goal over the next 3 years will be to enhance our insight of DRPLA clinical features, imaging and fluid biomarkers, disease progression, and to uncover methods to monitor response to therapeutic intervention.

## The next few years for DRPLA

The future for DRPLA and other rare disorders is one of momentous opportunity. The knowledge gained in the scientific community from previous successful (and many unsuccessful) trials for SMA, HD, and other similar diseases have defined the foundations required to understand disease progression and how to see the reversal. The current development of collaborative natural history and biomarker studies for DRPLA by our group at UCL alongside other institutions gives hope to DRPLA patients and families for advancements over the next few years. Whilst it is expected that many clinical, fluid or imaging markers of disease will overlap with other conditions, namely fluid NfL and MRI sequences, it can be postulated that DRPLA-specific markers may be discovered, for example, disease-associated protein levels such as ATN1, or somatic instability in the repeat expansion in biosamples. Though funding is challenging in rare disorders, natural history studies, in tandem with open-access data, imaging, wet biomarker and fibroblast repositories are essential. Ascertaining a wide resource for use by future researchers is crucial in the drive towards discoveries that may potentially benefit patient care.
