# Correction to: DRPLA: understanding the natural history and developing biomarkers to accelerate therapeutic trials in a globally rare repeat expansion disorder

**DOI:** 10.1007/s00415-021-10644-0

**Published:** 2021-06-21

**Authors:** Aiysha Chaudhry, Alkyoni Athanasiou-Fragkouli, Hector Garcia-Moreno, Silvia Prades Abadias, Julie Greenfield, Yael Shiloh-Malawsky, Paola Giunti, Henry Houlden

**Affiliations:** Department of Neuromuscular Disorders, UCL Institute of Neurology, Queen Square, London, WC1N 3BG UK

## Correction to: Journal of Neurology 10.1007/s00415-020-10218-6

The original version of this article unfortunately contained a mistake. The correct information is given below.

The name of the second author was misspelled, the correct name is given here: Alkyoni Athanasiou-Fragkouli.

Authors 3–7 were omitted during the original publication: Hector Garcia-Moreno, Silvia Prades Abadias, Julie Greenfield, Yael Shiloh-Malawsky, Paola Giunti.

Silvia Prades Abadias is the second correspondence author for this article jointly with Henry Houlden.

Author group should be:

Aiysha Chaudhry, Alkyoni Athanasiou-Fragkouli, Hector Garcia-Moreno, Silvia Prades Abadias, Julie Greenfield, Yael Shiloh-Malawsky, Paola Giunti and Henry Houlden

